# Expectation Modifies the Representational Fidelity of Complex Visual Objects

**DOI:** 10.1162/imag_a_00083

**Published:** 2024-02-02

**Authors:** Margaret Jane Moore, Amanda K. Robinson, Jason B. Mattingley

**Affiliations:** Queensland Brain Institute, University of Queensland, St. Lucia, Queensland

**Keywords:** predictive coding, EEG, multivariate pattern analysis, object representation, prediction

## Abstract

Prediction has been shown to play a fundamental role in facilitating efficient perception of simple visual features such as orientation and motion, but it remains unclear whether expectations modulate neural representations of more complex stimuli. Here, we addressed this issue by characterising patterns of brain activity evoked by two-dimensional images of familiar, real-world objects which were either expected or unexpected based on a preceding cue. Participants (n = 30) viewed stimuli in rapid serial visual presentation (RSVP) streams which contained both high-fidelity and degraded (diffeomorphically warped) object images. Multivariate pattern analyses of electroencephalography (EEG) data were used to quantify and compare the degree of information represented in neural activity when stimuli were random (unpredictable), expected, or unexpected. Degraded images elicited reduced representational fidelity relative to high-fidelity images. However, degraded images were represented with improved fidelity when they were presented in expected relative to random sequence positions; and stimuli in unexpected sequence positions yielded reduced representational fidelity relative to random presentations. Most notably, neural responses to unexpected stimuli contained information pertaining to the expected (but not presented) stimulus. Debriefing at the conclusion of the experiment revealed that participants were not aware of the relationship between cue and target stimuli within the RSVP streams, suggesting that the differences in stimulus decoding between conditions arose in the absence of explicit predictive knowledge. Our findings extend fundamental understanding of how the brain detects and employs predictive relationships to modulate high-level visual perception.

## 1 Introduction

Prediction plays a fundamental role in facilitating efficient and accurate visual perception (Bar, 2009; Rao & Ballard, 1999; Summerfield & Egner, 2009). Predictive coding models assert that during perception, bottom-up sensory input is compared with top-down predictions at multiple processing levels in the brain (Millidge et al., 2021; Summerfield & Egner, 2009). These comparisons are used to generate internal representations of stimuli which vary in their precision as a function of the comparative strength of, and agreement between, expectations and input (Millidge et al., 2021). Specifically, higher level brain areas such as the prefrontal and parietal cortices are thought to generate perceptual predictions, and these predictions are passed to lower-level sensory areas. These lower-level areas in turn generate prediction errors which are passed to higher levels and are used to refine stored models of the world. This effectively reduces information processing load since neural representations are only updated when new inputs deviate from what is predicted (Summerfield & Egner, 2009).

While predictive coding models have been influential in human vision science (Kok & de Lange, 2015), a number of key hypotheses arising from them remain untested. Specifically, although predictions have been shown to influence the encoding of low-level visual features, such as orientation and motion (Ekman et al., 2017; Hogendoorn & Burkitt, 2018; Tang et al., 2018), it is not known whether a similar influence arises for more complex visual stimuli which involve higher-order processes encompassing feature integration and extraction of meaning. To address this gap, here we recorded electroencephalography (EEG) while participants viewed statistically structured sequences of images depicting familiar real-world objects, and applied multivariate pattern analyses (MVPA) to determine whether predictability modulates how such objects are represented in the brain and whether predictions help resolve ambiguous perceptual input.

A key tenet of the predictive coding theory is that stimuli should be predictively encoded across multiple levels of the visual processing hierarchy (Kok & de Lange, 2015; Millidge et al., 2021). Previous studies in human observers and in animal models have shown that for elementary visual features such as orientation, unexpected stimuli yield enhanced representations relative to expected or random stimuli (Smout et al., 2019; Tang et al., 2018, 2023). However, it is unclear whether similar low-level predictive effects hold for complex, real-world stimuli which possess high-level semantic features. The potential role of predictive coding in modulating neural representations of higher-level visual stimuli has not been widely investigated. Using functional magnetic resonance imaging (fMRI) in human participants, Richter et al. (2018) found that neural responses to expected complex object stimuli were suppressed relative to those elicited by unexpected stimuli, across both early visual and higher-level ventral stream areas. Conversely, den Ouden et al. (2023) found no differences in face-evoked event-related potentials (ERPs) for expected versus unexpected stimuli. In a study of neuronal responses in the macaque inferior temporal cortex, Kaposvari et al. (2018) found that activity was enhanced for object stimuli that violated expectations relative to those that were expected.

Critically, most previous studies of visual prediction effects have compared differences in response magnitude for expected and unexpected stimuli, an approach which ignores potentially important stimulus-specific information contained within neural signals. More specifically, attenuation of signal-encoding for expected stimuli could plausibly result from either an overall lowering of stimulus-relevant activation, or from preserved stimulus-relevant activation coupled with a reduction in sensory noise (Feuerriegel et al., 2021). In line with the predictive coding theory, larger evoked responses to unexpected stimuli should reflect the additive effects of stimulus-relevant signals and prediction error (Summerfield & Egner, 2009). Conversely, elevated responses to unexpected stimuli may be unrelated to content-specific predictions, but might instead reflect the presence of surprise (see Feuerriegel et al., 2021). It is therefore important to quantify the information carried by neural signals generated in response to unexpected versus expected stimuli in order to further understanding of how predictive relationships affect visual perception.

The predictive coding theory also makes explicit assertions about how predictions modulate the encoding of sensory input. Specifically, top-down predictive signals are expected to constrain the interpretation of bottom-up visual input, thereby facilitating efficient (and accurate) interpretation of potentially degraded bottom-up sensory input (Bar, 2004; Summerfield & Egner, 2009). In other words, perceptual predictions should improve the precision with which degraded visual stimuli are encoded (Esterman & Yantis, 2010). Previous behavioural work has demonstrated that contextual and associative expectations facilitate recognition of visual stimuli (Esterman & Yantis, 2010; Joubert et al., 2007; Oliva & Torralba, 2007; Puri & Wojciulik, 2008), but little work has been undertaken to clarify the neural mechanisms underlying this effect.

Here, we addressed this issue by investigating how perceptual predictions modulate the neural encoding of both high-fidelity and perceptually degraded stimuli. We degraded our stimuli by applying diffeomorphic warping to a subset of the object images (for details, see Stojanoski & Cusack, 2013). Diffeomorphic degradation is designed to reduce identifying information while preserving low-level image characteristics, and has been shown to disrupt object recognition in human observers (Stojanoski & Cusack, 2013). We used diffeomorphic degradation to reduce the representational fidelity of object images, and asked whether object identification and associated neural activity are differentially affected when such degraded stimuli are expected versus unexpected.

Multivariate decoding analyses applied to time-resolved neuroimaging data provide an ideal method for evaluating these questions as they enable quantification of information encoded in the brain across time (T.A. Carlson et al., 2020; Oosterhof et al., 2016). Temporal multivariate pattern analysis (MVPA) is able to identify complex, multivariate relationships in patterns of activation across brain areas, and can be used to associate these variables with the information they represent (Mouchetant-Rostaing et al., 2000; Oosterhof et al., 2016). Here, we used multivariate decoding to quantify differences in the neural representations of complex object stimuli when these appeared in expected, unexpected, and random conditions. Our aim was to determine whether predictive information modulates the representation of both high-fidelity and degraded object images. We recorded participants’ brain activity using EEG as they viewed sequences of images depicting familiar real-world objects, and used MVPA to quantify whether, and to what extent, neural representations of identical objects are modulated by whether they are expected, unexpected or appear in the absence of any predictive structure.

## 2 Methods

### 2.1 Participants

Thirty participants (24 female, 2 left-handed, average age = 24.6 years, range = 20-52 years) were recruited from The University of Queensland and were compensated for their time at a rate of AUD 20 per hour. All included participants reported normal or corrected-to-normal vision and provided informed consent in writing. The study procedure was approved by The University of Queensland Human Research Ethics committee (HREA 2016001247).

### 2.2 Paradigm

**Fig. 1.**
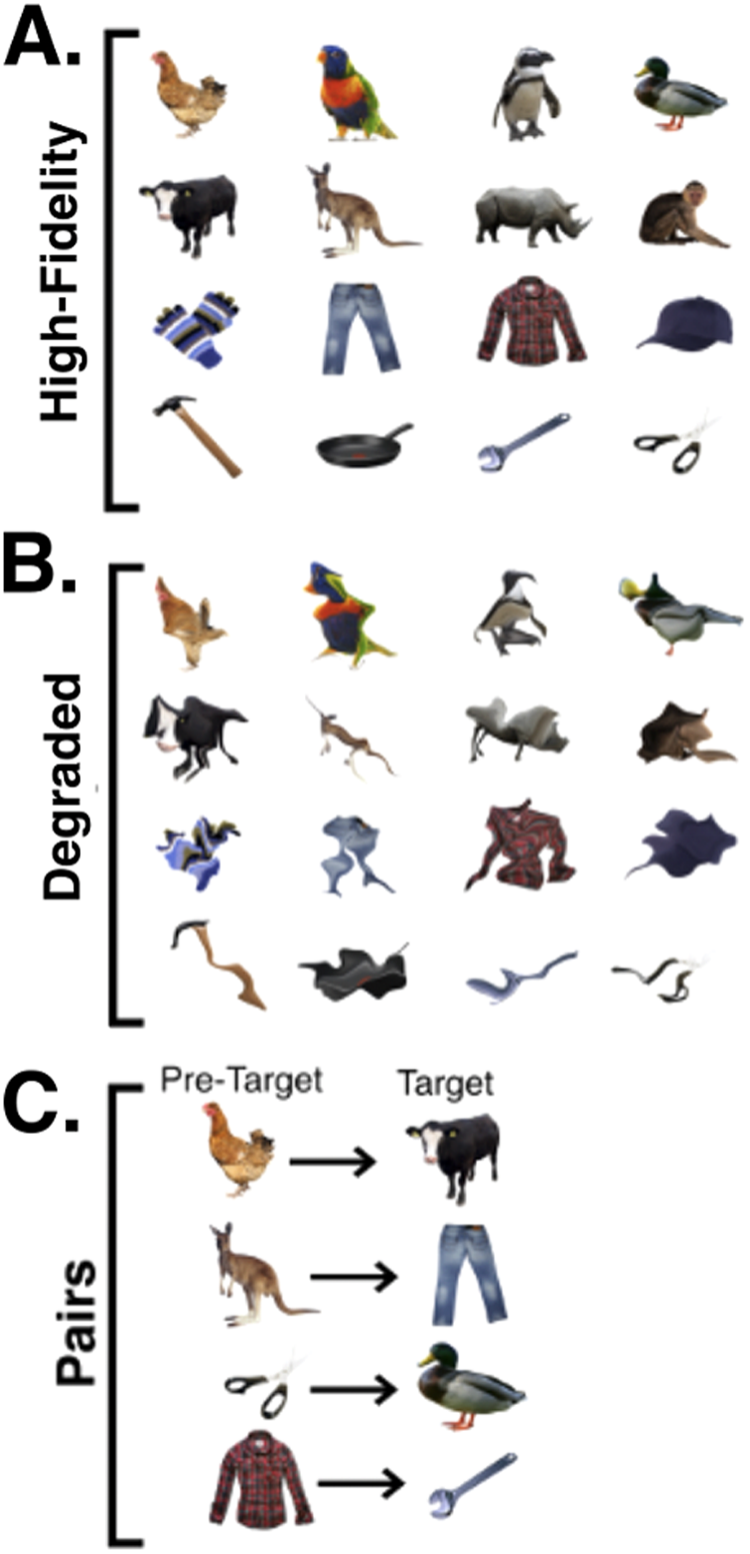
Visual stimuli used in the study. (A) High-fidelity images. (B) Degraded stimuli created through diffeomorphic warping. (C) Pre-target objects and their associated expected target stimuli. The same Pre-Target/Target pairs were used for all participants.

The study aimed to characterise neural representations of high-fidelity and degraded visual images of real-world objects that appeared at expected or unexpected positions within rapid serial visual presentation (RSVP) sequences at fixation. The stimulus images consisted of 16 objects obtained from www.pngimg.com. These images have been used in previous RSVP object-identity decoding paradigms, and are reliably decodable from EEG data (Grootswagers et al., 2019). Perceptual expectations were generated by introducing a reliable statistical structure to the order in which stimuli were presented. Specifically, image sequences were structured such that four pre-target images predicted the identity of a subsequent target stimulus with 80% accuracy. The same pre-target/target image pairs (defined in Fig. 1) were used for all participants. These target stimuli could be either high-fidelity or degraded. Degraded stimuli were constructed through diffeomorphic warping. This manipulation iteratively applies a flow field generated from cosine components with random phase and amplitude, leading to an equal probability of each pixel being expanded or contracted. The degraded stimuli were generated by applying seven sequential warps to images with a maximum possible distortion value of 20 (Stojanoski & Cusack, 2013).

**Fig. 2.**
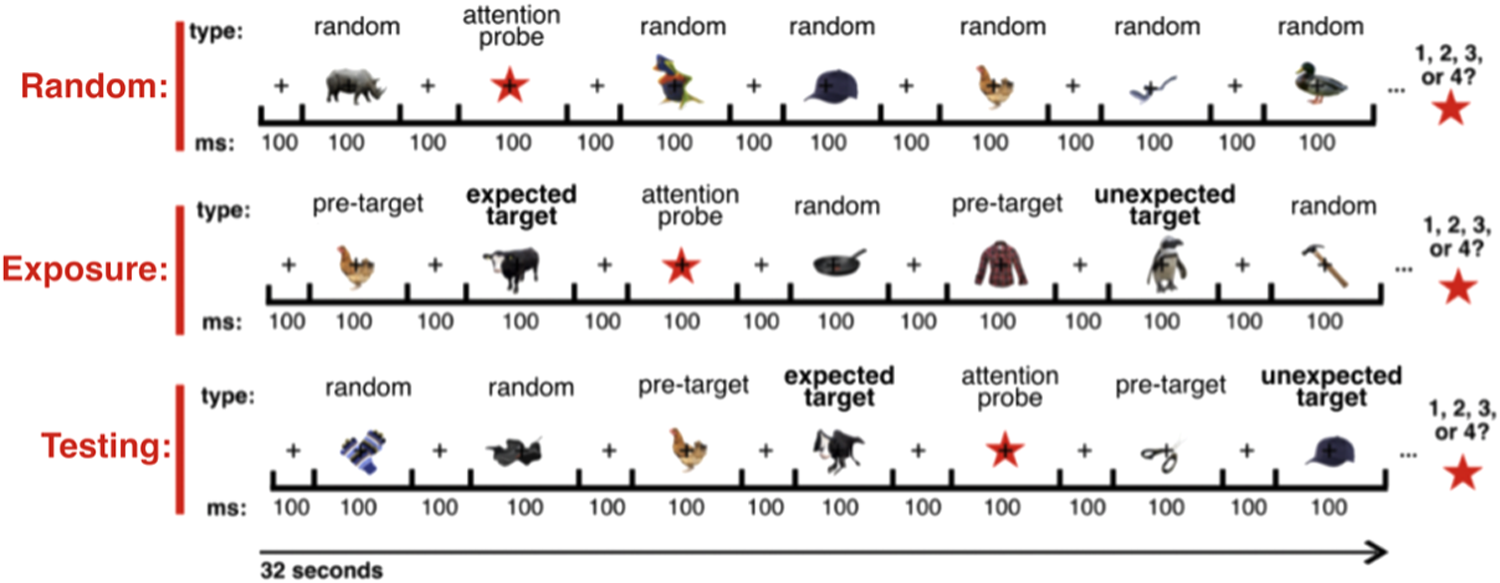
Examples of RSVP sequences included in each block type. Random Blocks contained all stimuli in random order. Exposure Blocks contained high-fidelity stimuli only and served to expose participants to the predictive structure of the RSVP sequences. Testing Blocks contained both high-fidelity and degraded stimuli in the described predictive structure. When a randomly presented (i.e., unpredictable) stimulus is presented following a pre-target stimulus, this random stimulus is classed as unexpected. Type denotes stimulus category, and ms refers to exposure time (in milliseconds).

RSVP streams were shown to participants in three distinct block types: Random, Exposure, and Testing (Fig. 2), as described below.

#### 2.2.1 Random block

The purpose of the Random block was to estimate robust neural representations for each object where no predictive information was present. In the Random Block, all 32 stimuli (both degraded and high-fidelity) were presented in random order with no statistical structure (8 sequences, ~40 exposures per unique stimulus images: high-fidelity or degraded).

#### 2.2.2 Exposure block

The purpose of the Exposure block was to expose participants to the predictive statistical structure within the RSVP streams. This exposure was intended to give participants an opportunity to passively learn (either explicitly or implicitly) stimulus associations prior to testing whether violating this structure modulated neural responses. In this block, high-fidelity stimuli were presented, and the predictive statistical structure was added (20 sequences, ~200 exposures per stimulus). The Exposure Block included an average of 200 exposures of each pre-target/target image pair, with 80% of pre-target images being followed by the “expected” stimulus and the remaining 20% being followed by an “unexpected stimulus” (see Fig. 1C). In the Exposure and subsequent Testing blocks, target stimuli were never presented in cases where they were not preceded by the appropriate pre-target stimulus (defined in Fig. 1). Unexpected stimuli were defined as any stimulus which appeared in a position in which a specific target was expected (see Fig. 2). Defined pre-target and target stimuli were never presented in unexpected positions.

Participants were not explicitly told of the predictive relationship between the pre-target/target pairs. Each predictive pair was presented 5-10 times per sequence. Data from the Exposure Block were used to passively expose participants to the used statistical structure only, and were not used to train or test decoding models. EEG data were recorded during the Exposure Block, but this was not used in any decoding analyses.

#### 2.2.3 Testing block

The purpose of the Testing Block was to generate the data needed to evaluate whether predicted versus unpredicted targets yielded different neural decoding accuracies relative to the same stimuli presented randomly (i.e., in which there was no predictive cue to the targets’ occurrence). The Testing Block included 45 RSVP sequences (~450 exposures per predictive pair). Both degraded and high-fidelity stimuli were presented within the Testing Block. In this block, 40% of pre-target images were followed by the high-fidelity expected stimulus, 40% were followed by the degraded version of the expected stimulus, and 20% were followed by an unexpected object (objects never predicted). Testing Block images were classed as either (1) “expected,” in which the target stimuli were consistent with the identity predicted by the preceding pre-target stimulus; (2) “unexpected,” where the target stimuli were inconsistent with the identity predicted by the pre-target; or (3) random, in which there was no predictive information carried by the preceding image. These designations were assigned to both the high-fidelity and degraded stimuli.

Participants were instructed to monitor the RSVP sequences and perform an orthogonal task in which they reported the number of attention probes (red stars) present in each sequence. The goal of the attention task was to ensure participants maintained attention on the stimulus streams without the requirement to perform explicit object identification (Grootswagers et al., 2019). Stimuli were presented at a rate of 5 Hz (100 ms exposure with 100 ms blank interstimulus intervals). Participants viewed 73 RSVP sequences, each containing 160 object stimuli and 1-4 attention probes. Attention probes were pseudorandomly distributed with the constraint that they could not appear within the first or last 10 images, and that a minimum of 10 images were shown between each attention probe. Sequences were also constructed such that no image was immediately repeated. At the end of each stream, participants were prompted to report how many attention probes they had seen using numbered keys (1-4), and were provided feedback on their accuracy.

The full paradigm lasted approximately 60 minutes, and participants were encouraged to take short rest breaks between sequences. Following the main experiment, participants completed a debrief questionnaire, the aim of which was to assess whether they were aware of the statistical structure embedded within the relevant blocks. Participants were first asked to report whether they “noticed anything about the images.” Next, they were asked to freely report whether they “noticed any patterns within the images.” Following these free response questions, participants were informed that the presented sequences contained some images which predicted the identity of the subsequent image, and were given multiple-choice questions to assess whether they were able to identify the pre-target and target images. Following this, participants were shown each of the four pre-target images in turn and asked to indicate its associated target image, guessing if necessary.

### 2.3 EEG recordings and pre-processing

Continuous EEG data were recorded using a BioSemi system and digitized at a sampling rate of 1024 Hz. The 64 electrodes were arranged according to the international standard 10–10 system for electrode placement (Oostenveld & Praamstra, 2001). Recorded data were pre-processed using EEGLAB functions (Delorme & Makeig, 2004). Specifically, raw EEG data were re-referenced to mastoid channels and were filtered using high pass (0.1 Hz) and low pass (100 Hz) frequency filters. Noisy electrode channels were identified using joint probability, and channels were rejected if they exceeded 5 standard deviations from the average (mean number interpolated = 1.07 electrodes, SD = 1.89, range = 0-6). These channels were reconstructed using spherical interpolation. EEG data were then down-sampled to 256 Hz, divided into stimulus presentation-locked epochs including the time interval from [-100 ms to +1000 ms] from stimulus presentation, and baseline corrected. No other pre-processing or data cleaning was performed.

### 2.4 Decoding analysis

EEG data were analysed using a multivariate pattern analysis (MVPA) decoding pipeline (Grootswagers et al., 2017; Oosterhof et al., 2016) involving regularised linear discriminant analysis (LDA) based classifiers. Importantly, this method quantifies the discriminability of images from the neural signal, essentially measuring the degree of information carried by neural signals rather than simply detecting differences in signal magnitude. Decoding analyses were preformed based on raw EEG voltages across all scalp electrodes. Decoding was preformed for each timepoint independently, and additional temporal data were not considered in analysis. Decoding analyses were implemented using CoSMoMVPA (Oosterhof et al., 2016) in MATLAB. All decoding analyses were performed at the participant level, but overall results were analysed at the group-level.

For each participant, pair-wise decoding was conducted across all object pairs present in the condition of interest. For each pair of objects (objects A and B), decoding classifiers only considered data from epochs in which object A or B was presented. Classifiers were trained to distinguish image A from image B based on 80% (randomly selected) of the included epochs, and the accuracy of this model was assessed by calculating the model’s percent correct when used to distinguish between A and B in the remaining randomly selected 20% of relevant epochs. If classifiers are unable to reliably distinguish between A and B in the testing dataset, model accuracy will be at chance (50%). This process is repeated five times for each pair, with accuracy being the average percent correct classifications within the testing data. This process is repeated independently across each timepoint for each of the included object pairs. Overall decoding accuracy for each condition is the average accuracy of classifiers across all considered object pairs in a single participant. This accuracy data is averaged at a group-level to yeild final decoding accuracy values.

In each analysis, all pairwise combinations of images which were presented in the relevant condition (i.e., cow versus shirt, cow versus duck, etc.) were decoded. The exact number and identity of object pairs in each analysis is determined by the condition being considered. For example, the four potentially expected objects were considered in analyses aiming to decode expected objects while only the eight potentially unexpected objects were included in analyses decoding unexpected objects (see Figs. 1 and 2). Each decoding analysis involved training classifiers on images presented in the independent Random Blocks, in which there was no statistical structure in the ordering of images and testing the classifier model on the same images from specific conditions of interest in the Testing Block. These models formed the basis for comparing object representations across all conditions. For assessing representations in the random condition, the models were tested on the remaining epochs of the same condition per iteration (20%). In cases where models were trained and tested on different stimulus types (e.g., trained on high-fidelity images, tested on degraded images) or different conditions (e.g., trained on random, tested on the predicted), the identical epochs and classification schemes were used to train the classifier, but models were tested on data from another condition. For example, the exact training model indices used in the model trained to distinguish the identities of high-fidelity stimuli was tested on data from epochs in which degraded stimuli were presented. The number of trials included in each decoding model is reported in Supplementary Table 1. Due to the pairwise classification scheme, chance decoding accuracy was 50%.

### 2.5 Statistical inference

In each decoding analysis, statistical testing was conducted to determine whether stimulus information was present in the relevant EEG signal. To determine whether decoding accuracy was above chance and to quantify differences in decoding accuracies across conditions, Bayes factors (BF) were computed using the R package BayesFactor (Dienes, 2011; Rouder et al., 2009; Teichmann, 2022). These analyses employed alternative hypotheses with JZS prior (default scale factor = 0.707) and a null hypothesis prior set at chance level. Bayes factors were then calculated to represent the probability of the observed data occurring under the alternative hypothesis relative to the null hypothesis. All in-house scripts for using Bayes factor analyses to detect differences in decoding accuracy are openly available (https://osf.io/cqyp2/). In line with standard interpretation guidelines, Bayes Factors >10 were interpreted as strong evidence in support of the alternative hypothesis, and Bayes Factors <1/3 represented strong evidence in favour of the null hypothesis (Jarosz & Wiley, 2014; Rouder et al., 2009). Bayes Factors >3 and <10 were interpreted as representing moderate evidence in support of the alternative hypothesis. We designated the onset of decoding as the timepoint at which there was moderate evidence (BF > 3) of above-chance classifier performance over at least three successive time points. In cases where this criterion was not met, decoding accuracy (or differences between decoding models) was not considered to be reliable.

Frequentist cluster-based permutation corrections for multiple comparisons are also reported. For these corrections, t-tests were conducted at each timepoint (one-tailed for vs. chance comparisons, two-tailed for between-model comparisons). Analyses yielding p-values <0.01 were included in clusters. For each comparison, 10,000 permutations were conducted to calculate the probability that each defined cluster (summarised by the cluster t-score sum) could occur when no underlying effect was present. All clusters with resultant p-values of <0.05 are reported.

## 3 Results

### 3.1 Behavioural analyses

All participants performed above chance accuracy (25%) on the attention-probe task, with an average accuracy of 88.1% (SD = 9.67, range = 47%–99%). In the debrief questionnaires, no participants spontaneously reported noticing any patterns in the presented stimuli. In free-response multiple choice questions, four participants correctly reported one of the four employed stimulus pairs. In prompted matching, six participants correctly identified one of the four employed stimulus pairs. Each correct response was provided by a different participant. All participants who correctly reported pre-target/target pairs in the free-response multiple choice failed to replicate these correct responses within the multiple-choice questions. Overall performance on both free-response multiple choice and prompted matching was not significantly different from chance (free-response: X^2^(1) = 0.062, p = 0.804; prompted: X^2^(1) = 0.212, p = 0.645), indicating that participants were not explicitly aware of the predictive structure present within the RSVP sequences.

### 3.2 Decoding analysis 1: effect of diffeomorphic degradation on image classification fidelity

**Fig. 3.**
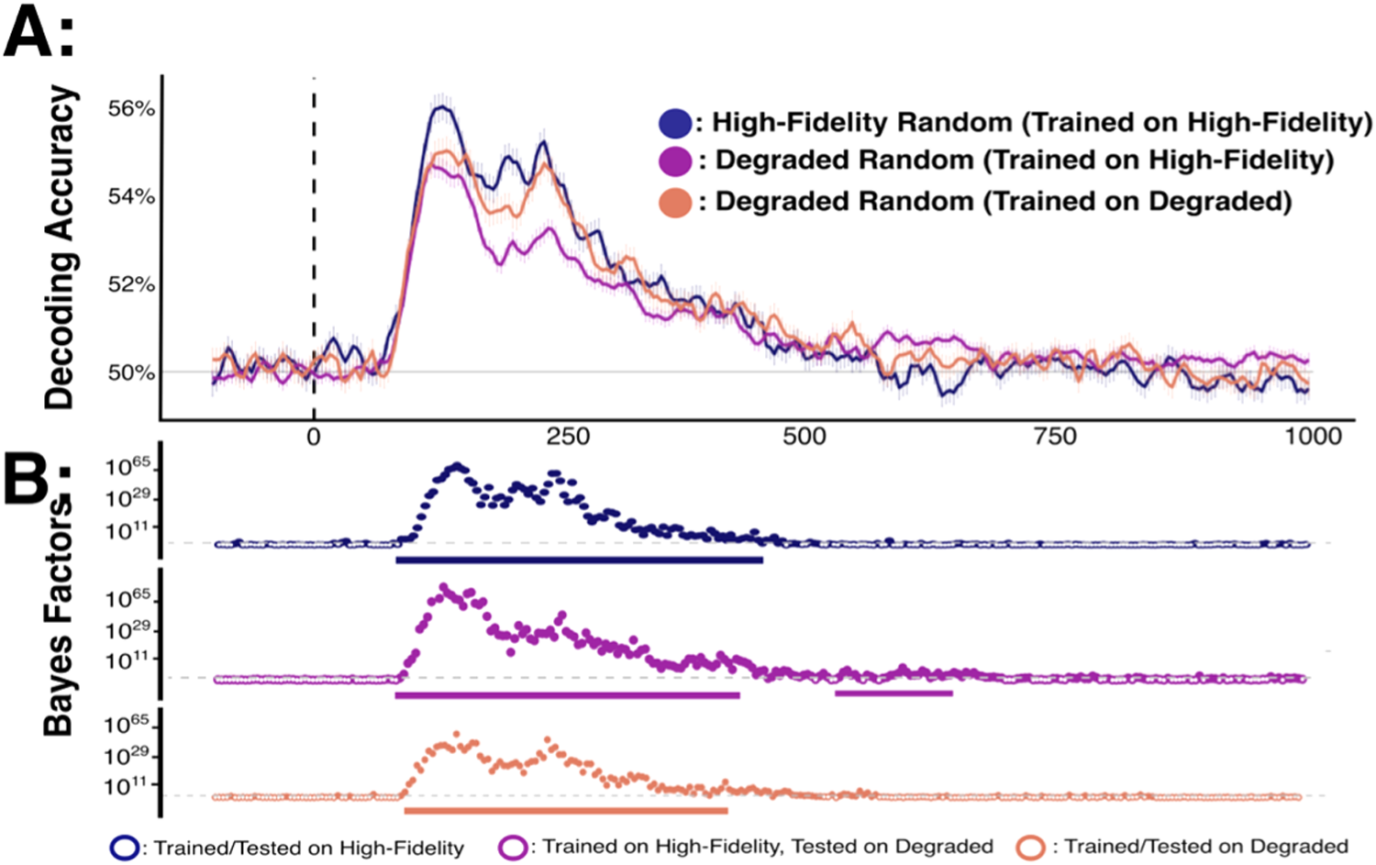
Decoding accuracy for randomly presented high-fidelity and degraded stimuli for 16 different object identities. (A) Sample mean decoding accuracy (upper) and BF comparisons between models (lower) across time (x-axis in ms). Chance performance (50%) is denoted in grey, and stimulus onset is denoted by the dotted vertical line. (B) Bayes Factor plots. In these plots, the dotted grey line marks BF = 3 (the boundary for moderate evidence). Tests yielding BF > 3 are represented by filled dots. Solid bars beneath Bayes factor plots highlight comparisons which survive frequentist cluster-based permutation corrections for multiple comparisons (p < 0.05).

In a first step, the representations of high-fidelity and degraded stimuli shown in the Random RSVP sequences were compared (Fig. 3). In this analysis, a model was trained and tested on high-fidelity stimuli presented in the Random Block, and then tested on both high-fidelity and degraded stimuli presented within the same block. This analysis aimed to establish baseline representational differences between degraded and high-fidelity stimuli in the absence of predictive structure. As a control, a classifier was also trained and tested on degraded stimuli from Random RSVP sequences (Suplementary Figure 1).

We designated the onset of decoding as the timepoint at which there was moderate evidence (BF>3) of above-chance classifier performance over at least three successive time points. Decoding accuracy for degraded stimuli was significantly lower than decoding accuracy for high-fidelity stimuli. Specifically, decoding accuracy was consistently above chance for both high-fidelity (from 78–457 ms) and degraded stimuli (from 82–500 ms). This indicates that patterns of neural activity reliably distinguished between object identities in the absence of any statistical structure in the RSVP sequences. Critically, decoding accuracy for degraded stimuli was consistently lower than that of high-fidelity stimuli between 117-140 ms and 164-290 ms following stimulus presentation.

### 3.3 Decoding analysis 2: effect of fulfilled predictions on image classification fidelity

**Fig. 4.**
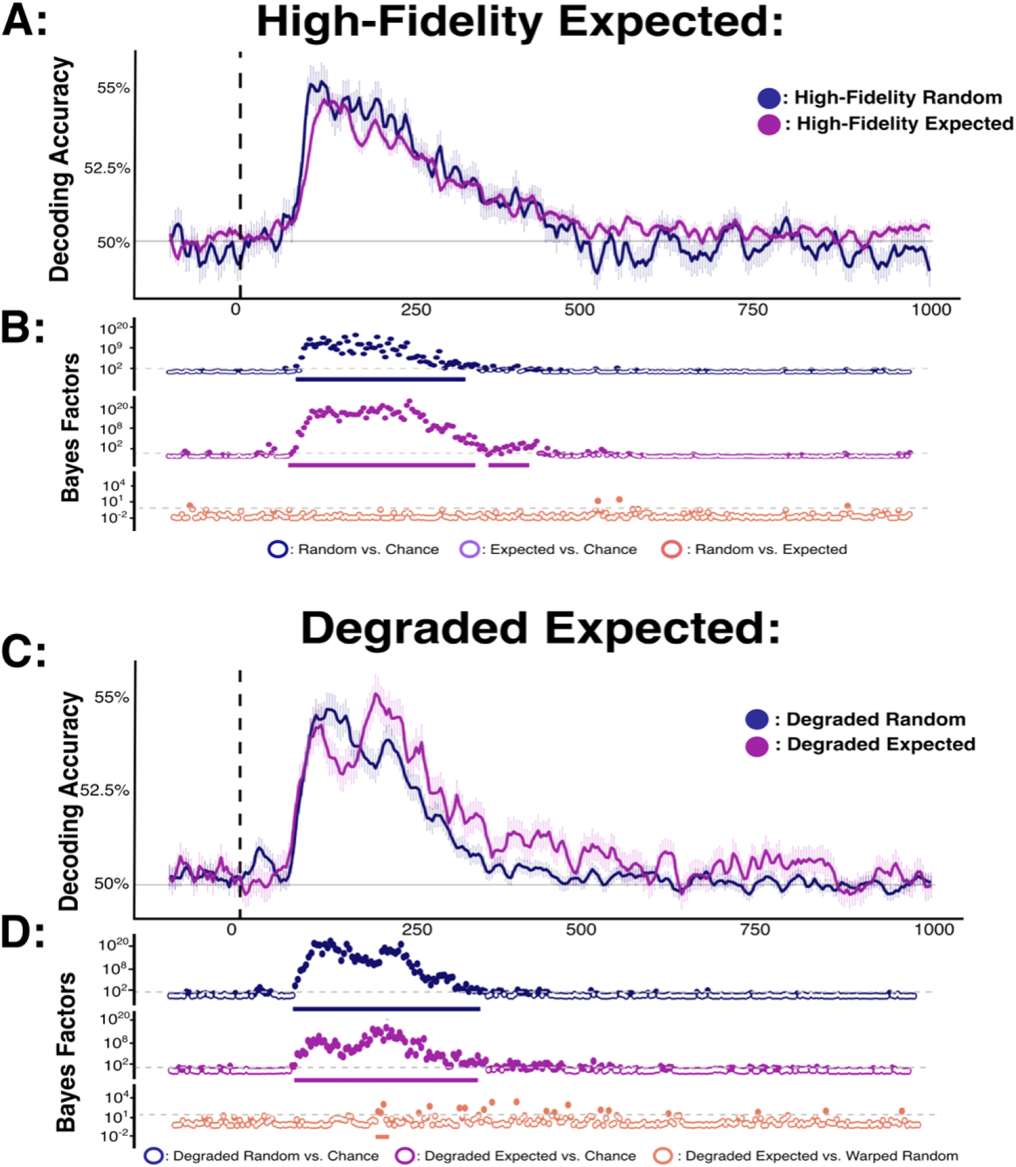
Decoding accuracy for randomly presented versus expected stimuli. (A, C) Decoding accuracy for high-fidelity and degraded stimuli, respectively. In these panels, the sample mean decoding accuracy is shown for all pair-wise stimulus combinations of the four possible expected stimuli across time (x-axis in ms). Chance performance (50%) is denoted in grey, and stimulus onset is denoted by the dotted vertical line. (B, D) Bayes Factor plots for each decoding model comparison. Within the Bayes Factor plots, the dotted grey line marks BF = 3 (the boundary for moderate evidence). Dots which are coloured represent comparisons yielding BF > 3. Solid bars beneath Bayes factor plots highlight comparisons which survive frequentist cluster-based permutation corrections for multiple comparisons (p < 0.05).

In a second step, we tested for representational differences between identical objects that were either expected (thus fulfilling predictions) or random (in which no specific object identity could be predicted; see Fig. 4). We initially considered only high-fidelity stimuli. We trained a classifier on high-fidelity stimuli from the Random block and compared decoding accuracy for each relevant object when it appeared predictably or randomly. Decoding accuracy for predicted stimuli was consistently above chance from 78-429 ms after stimulus onset, but there was minimal evidence for a difference in decoding accuracy for predicted versus random stimuli (max BF = 1.78 at 199 ms). Next, we compared decoding accuracy for degraded stimuli across predicted and random presentations. For this analysis we trained the classifier on high-fidelity stimuli from the Random block and examined decoding accuracy for each degraded object when it appeared predictably or randomly. Decoding accuracy was above chance for degraded stimuli in both the random and predicted conditions, indicating separable representations for these images. Notably, decoding accuracy for degraded stimuli was reliably higher for predicted relative to random objects at 13 timepoints between 191 and 464 ms, indicating that the degraded objects had an enhanced neural representation when their high-fidelity versions were expected.

### 3.4 Decoding analysis 3: effect of violated predictions on image classification fidelity

**Fig. 5.**
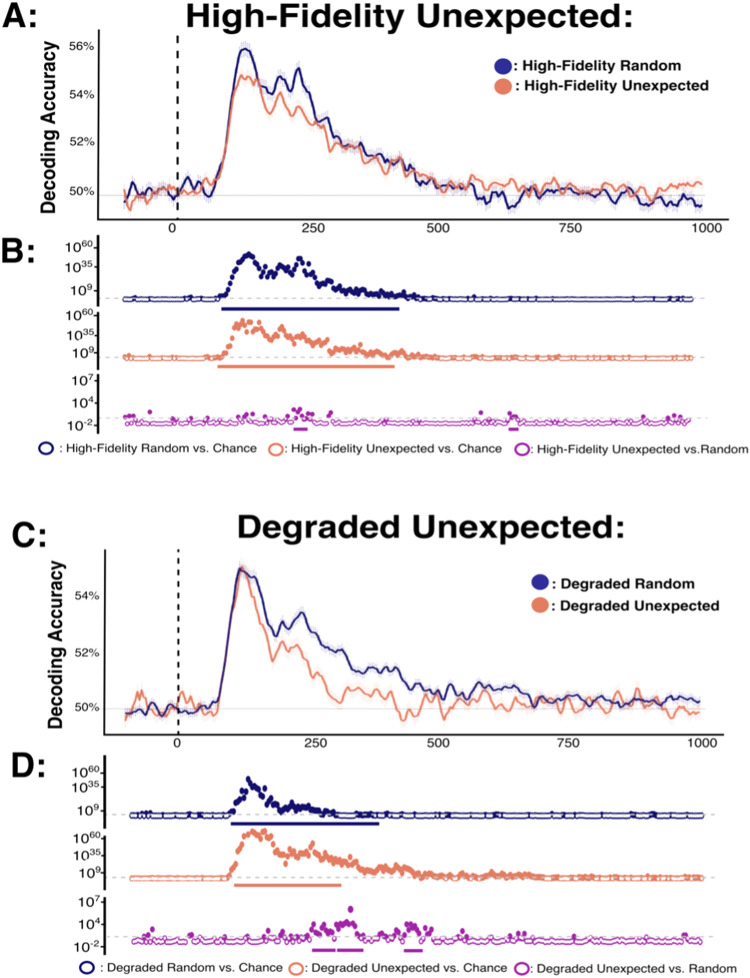
Comparison of decoding accuracies for high-fidelity stimuli presented in random versus unexpected positions. (A, C) Decoding accuracy for high-fidelity and degraded stimuli, respectively. Panels show sample mean decoding accuracy for all pair-wise stimulus combinations of the eight possible unexpected stimuli across time (x-axis in ms). Chance performance (50%) is denoted in grey, and stimulus onset is denoted by the dotted vertical line. (B, D) Bayes factors for each relevant decoding model comparison. Dotted grey lines mark BF = 3 (the boundary for moderate evidence). Dots which are coloured represent comparison yielding BF > 3. Solid bars beneath Bayes factor plots highlight comparisons which survive frequentist cluster-based permutation corrections for multiple comparisons (p < 0.05).

In a third set of analyses, we tested for representational differences between identical objects that were random or unexpected (see Fig. 5), separately for high-fidelity and degraded stimuli, using an analogous approach to that described above for predicted stimuli. For high-fidelity stimuli, decoding accuracy for unexpected objects was consistently above chance between 82-476 ms after stimulus onset. Likewise, for degraded stimuli decoding accuracy for unexpected objects was consistently above chance from 82-273 ms. Critically, there were also reliable differences in decoding between identical objects that were unexpected versus random. Specifically, for high-fidelity stimuli decoding accuracy for unexpected objects was intermittently lower than that of random objects between 113-164 ms and 207-289 ms post-target onset. This difference in pair-wide decoding accuracy was generally consistent across the individual image comparisons (Supplementary Fig. 2). Likewise, for degraded stimuli decoding accuracy for unexpected objects was consistently below that of random objects between 234-324 ms.

### 3.5 Decoding analysis 4: decoding representations of prediction content

**Fig. 6.**
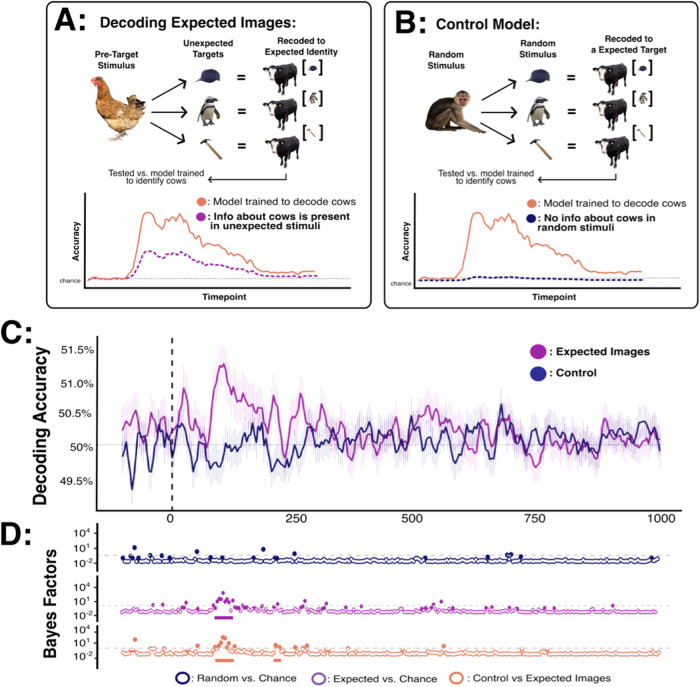
Decoding the identity of expected (but not presented) stimuli. (A) In the test model, the identity of expected target stimuli was decoded from trials in which these stimuli were expected, but not presented. (B) In the control model, the identity of expected target stimuli was decoded from random trials in which no stimuli were expected. These models were tested against a classifier trained to distinguish between expected stimuli (e.g., the model used in Fig. 4). If the tested stimuli do not contain any information about the expected (but not presented) image, decoding accuracy should be at chance. However, if the stimuli do contain information pertaining to the expected image, decoding accuracy should be above chance. (C) Decoding accuracy for all pair-wise stimulus combinations across time (x-axis in ms). Chance performance (50%) is denoted in grey, and stimulus onset is denoted by the dotted vertical line. (D) Bayes factors for each relevant comparison across time. The dotted grey line marks BF = 3 (the boundary for moderate evidence). Dots which are coloured represent comparison yielding BF > 3. Solid bars beneath Bayes factor plots highlight comparisons which survive frequentist cluster-based permutation corrections for multiple comparisons (p < 0.05).

In a final step, we asked whether neural representations of expected stimuli could be decoded from patterns of brain activity even when the expectation was violated (i.e., when an unexpected stimulus was shown instead). To do this, all trials containing an unexpected stimulus (both high-fidelity and degraded) were tested using a model trained to identify stimuli that were expected. Critically, this comparison aimed to decode information about the stimulus that was expected, *but not presented* (see Fig. 6). To ensure decoding performance was not related to low- or high-level similarities between expected and unexpected stimuli, a control analysis was also conducted. Specifically, the same decoding models were tested on data matched to the comparator analysis in terms of stimulus identity, expected stimulus identity, trial numbers, and block of acquisition. The only difference between these main and control analyses was that in the main analysis, specific stimuli were expected (but not presented), whereas in the control analysis specific stimuli were neither expected nor unexpected (i.e., they appeared randomly).

As shown in Figure 6, decoding accuracy for expected (but not presented) stimuli was above chance from 80-360 ms after stimulus onset. By contrast, for the control analysis decoding accuracy was not consistently different from chance between 0 and 500 ms (max BF = 3.49). Critically, decoding accuracy for expected (but not presented) stimuli was consistently above that of the control model between 84 and 348 ms. While the peak decoding accuracy for expected (but not presented) stimuli was lower than that shown in Figure 4 for expected (and presented) stimuli, this is not surprising given that the decoded stimulus in the former was never actually presented. Although here we combined data from both high-fidelity and degraded stimuli, trends towards the reported decoding effects were also present when the analysis was repeated for high-fidelity and degraded stimuli separately (see Supplementary Fig. 3).

## 4 Discussion

The goal of our study was to determine whether visual images of real-world objects are represented differently in the brain when the likelihood of their occurrence (i.e., their predictability) is systematically varied. Previous work using low-level visual properties has shown that unexpected stimuli are represented with higher fidelity than those which are expected or appear with no predictive information (i.e., at random) (Smout et al., 2019; Tang et al., 2018). To date, however, no study has investigated the influence of expectations on multivariate representations of higher-level visual objects. We found that neural representations of identical object stimuli were modulated by expectation. In contrast to previous investigations (Smout et al., 2019; Tang et al., 2018), a significant reduction was identified in the information represented for unexpected stimuli relative to random stimuli. We also found evidence of co-activation of expected and unexpected representations in cases where a specific image was expected but was not presented. Notably, neural representations of degraded, but not high-fidelity, stimuli were enhanced when objects were predicted, indicating that expectation influences the fidelity of neural patterns of activity related to ambiguous visual input. Participants were not explicitly aware of this predictive structure and key prediction effects were in the opposite direction as would be expected if they were driven by general surprise or attention effects (e.g., worse decoding for stimuli expected to cause surprise or capture attention) (Alink & Blank, 2021; Feuerriegel et al., 2021; Grootswagers et al., 2021).

Critically, predictive information modulated neural repesentations of presented images. For the degraded images, expected stimuli yielded higher decoding accuracies than when identical stimuli were presented randomly. This finding indicates that predictive relationships may help resolve perceptual uncertainty and facilitate higher-fidelity representations of degraded visual stimuli. Interestingly, these effects were not evident for high-fidelity objects: decoding accuracy for high-fidelity images was not different in cases where these stimuli were expected versus random. Future research is needed to evaluate whether this null result indicates that predictive information mainly contributes to uncertainty resolution or whether prediction subtly modulates the representations of high-fidelity stimuli in a manner which was not detected in this paradigm.

Additionally, we found that unexpected stimuli have reduced representational fidelity compared with identical, randomly presented images. In a previous study, Tang et al. (2018) found that orientation selectivity was increased for unexpected relative to expected gratings. This result apparently contrasts with those of the present study, but there are several potential explanations for this difference. First, the content of the generated predictions likely varied across studies. In the study of Tang et al. (2018), the generated predictions contained information specifically pertaining to stimulus orientation. While similar low-level feature predictions could plausibly be generated by the statistical structure used in this study, low-level predictions are not strictly necessary to explain the results of the present study. For example, when a cow is expected, this prediction could be operationalised in terms of expecting any image with cow-like semantic content (e.g., a prototypical cow image, or the same cow from any angle) instead of activating a specific pattern of expected low-level features (as in Tang et al., 2018).

This possibility is supported by our findings that predictive effects mainly arose in the time window associated with representations of categorical and semantic object information (peak latency at 150 – 250 ms) (T. Carlson et al., 2013; Cichy et al., 2016). For this reason, the current results build upon rather than conflict with the findings of previous studies examining the impact of low-level feature predictions. The current study provides evidence that predictions modulate how object stimuli are represented, but additional work is needed to clarify the exact content of these predictions and the specific perceptual stages at which they are integrated. It is therefore important for future studies to more explicitly investigate the effects of predictions for high- and low-level features within a single task to examine whether predictions for these features are independent or integrated.

Notably, in some cases, predictive effects continued after 250 ms post-presentation. This later time window is outside the range generally associated with object recognition processes but aligns with the timing of more complex visual processing (e.g., natural and effective scene processing) (Bo et al., 2022; Hansen et al., 2021). In line with predictive coding framework, these later differences in signal fidelity might plausibly represent the encoding, transmission, and integration of prediction error (or prediction updating across the visual hierarchy (Summerfield & Egner, 2009). This is because prediction error/updating signalling occurs following the initial detection of a predictive relationship and would result in differences in signal information relative to the random condition. This change could be expected to result in decoding accuracy differences at later timepoints following the detection of predictive information.

In cases where expectations were violated, the observed neural responses contained content-specific information about the expected stimulus, and content-specific information about the presented stimulus*.* This result is in line with past work. Specifically, Smout et al. (2019) found that neural responses to unexpected grating stimuli contained information related to the difference between expected and presented (unexpected) stimulus orientations. Our study builds upon this finding by demonstrating that neural responses to unexpected object stimuli also contain information about the stimulus which was expected (but not presented) in addition to visual information about the presented stimulus. Such activation of multiple representations concurrently might explain the comparatively lower decoding performance for unexpected stimuli, as these conflicting representations would likely result in noisier patterns of evoked neural activity.

This current study also aimed to investigate differences in representational dynamics between degraded and high-fidelity versions of the same object stimuli. The diffeomorphic warping we used is designed to preserve early visual features while disrupting higher recognition-related image information (Stojanoski & Cusack, 2013). Our findings suggest that the very early representations of high-fidelity and diffeomorphically transformed stimuli are indeed similar, but that differences emerge before high-level stimulus properties are encoded.

Considered cumulatively, the findings of the current study provide an important and novel test of the key hypotheses of the predictive coding theory. First, the results demonstrate that neural representations of expected and unexpected object images are inherently different. This difference does not simply correlate with differences in surprise, attention, or response magnitude, but instead represents a difference in the type and quality of information represented in multivariate patterns of brain activity. Second, the identified differences in decoding accuracy at timepoints associated with the representation of categorical semantic information provide evidence of predictive modulation within representations of high-level visual features. This result provides neurophysiological support for a key hypothesis arising from the predictive coding theory, namely, that information is predictively encoded throughout the visual processing hierarchy. Our study also suggests that predictive relationships modulate how information about ambiguous visual stimuli is encoded, demonstrating that expectations can be employed to facilitate more effective recognition in cases where input is uncertain. Finally, our results provide evidence that representations of specific complex stimuli are activated in response to perceptual predictions, regardless of whether these expected stimuli actually appear.

## 5 Conclusion

Here we have provided novel insight into how predictive relationships modulate neural representations of complex visual objects. Predictive structure was found to help resolve perceptual uncertainty, even in cases where participants were not explicitly aware of this structure. Finally, representations of expected stimuli were activated in cases where specific images were expected but were not presented. Taken together, the current findings extend fundamental understanding of how the human brain detects and employs predictive relationships to modulate visual perception.

## Supplementary Material

Supplementary Material

## Data Availability

All data, paradigm materials, and analysis code associated with this project are openly available on the Open Science Framework (https://osf.io/cqyp2/).
