# Supplementary material for: Expectation Modifies the Representational Fidelity of Complex Visual Objects
Source: Imaging Neurosci (Camb). 2024 Feb 2;2:imag-2-00083. doi: 10.1162/imag_a_00083 (PMC12224411; doi:10.1162/imag_a_00083)
Supplement: Supplementary Material [file imag_a_00083-supp.pdf]

Supplemental Materials:

Supplementary Table 1: *Summary of the 10 reported decoding analyses. 'Training Data' corresponds to the stimulus types used to train the classifier, whereas 'Testing Data' refer to the stimulus types used to test the model. Where testing data are not reported, models were trained and tested on the same stimuli. 'N Epochs' refers to the number of epochs (per participant) used to train or test each classifier. 'Onset' indicates the first time at which at least three sequential timepoints were above chance (BF > 3). 'End' refers to the last timepoint at which at least three sequential timepoints were above chance (BF > 3). 'Peak Accuracy' is*

Training Data	Testing Data	Figure	N Epochs	Onset	End	Peak Accuracy	Peak Time	N BF > 3
High-fidelity Random (Control)		3	287 (40.3, 230-333)	78	445	56.5%%	125	100
High-fidelity Random (Expected)		4	142 (18.3, 120-169)	90	344	56.1%%	121	73
High-fidelity Random (Control)	Degraded Random (Control)	3	269 (57.8, 187 - 325)	82	484	55.1%%	113	149
High-fidelity Random (Expected)	Degraded Random (Expected)	4	112.6 (10.1, 94-120)	74	355	55.30%	199	97
High-fidelity Random (Expected)	High-fidelity Expected	4	575 (34.1, 470-618)	78	430	55.7%%	129	106
High-fidelity Random (Expected)	Degraded Expected	4	578 (38.3, 465-623)	78	352	54.9%%	152	81
High-fidelity Random (Control)	High-fidelity Unexpected	5	128.1 (12.7, 99 - 150)	82	477	55.4%%	121	117
High-fidelity Random (Control)	Degraded Unexpected	5	125.1 (14.0, 96-155)	82	277	55.2%%	117	64
High-fidelity Random (Control)	Unexpected (recoded as Expected)	6	253.2 (22.2, 201-298)	89	113	51.4%%	102	18
High-fidelity Random (Control)	Random (Recoded as Expected)	6	253.2 (22.2, 201-298)	NA	NA	51.00%	180	6

the maximum mean classifier accuracy, and 'Peak Time' is the time at which this occurred. 'N BF > 3' reports the number of timepoints at which decoding accuracy was above chance (BF > 3) between -100 and 1000ms from stimulus onset.

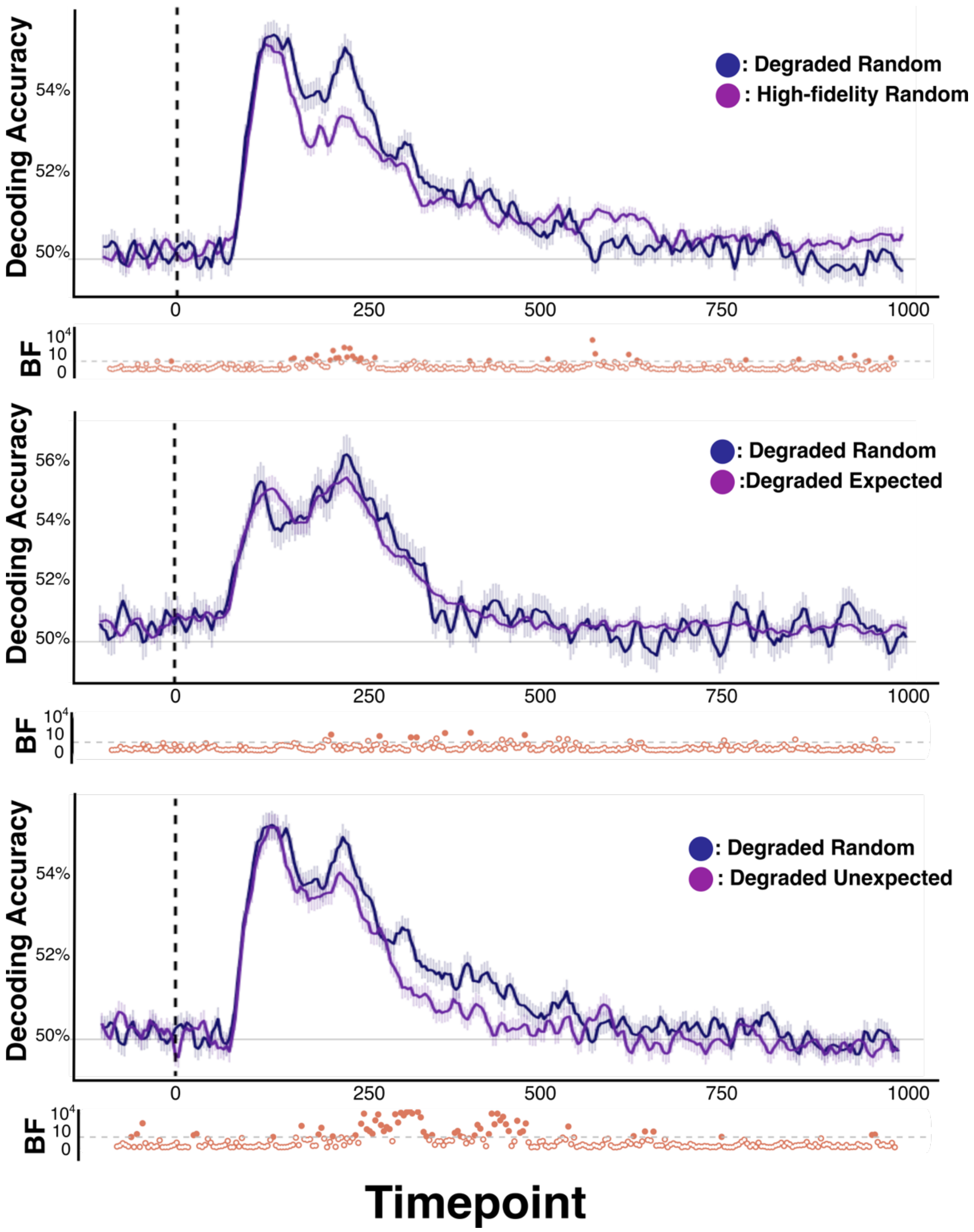

Supplementary Figure 1: *Decoding accuracy for the reported expectation analyses when classifiers were trained on data from degraded random stimuli and tested on high-fidelity random (upper panel), degraded-expected (centre panel), and degraded-unexpected stimuli (lower panel), respectively. Chance performance (50%) is denoted in grey and stimulus onset is denoted by the dotted vertical line. Bayes factors for each relevant decoding model comparison are reported in lower panels. Dotted grey lines mark BF = 3 (the boundary for moderate evidence). Dots which are coloured represent comparison yielding BF >3. These results are similar to those found when classifiers were trained on high-fidelity stimuli and tested on degraded stimuli.*

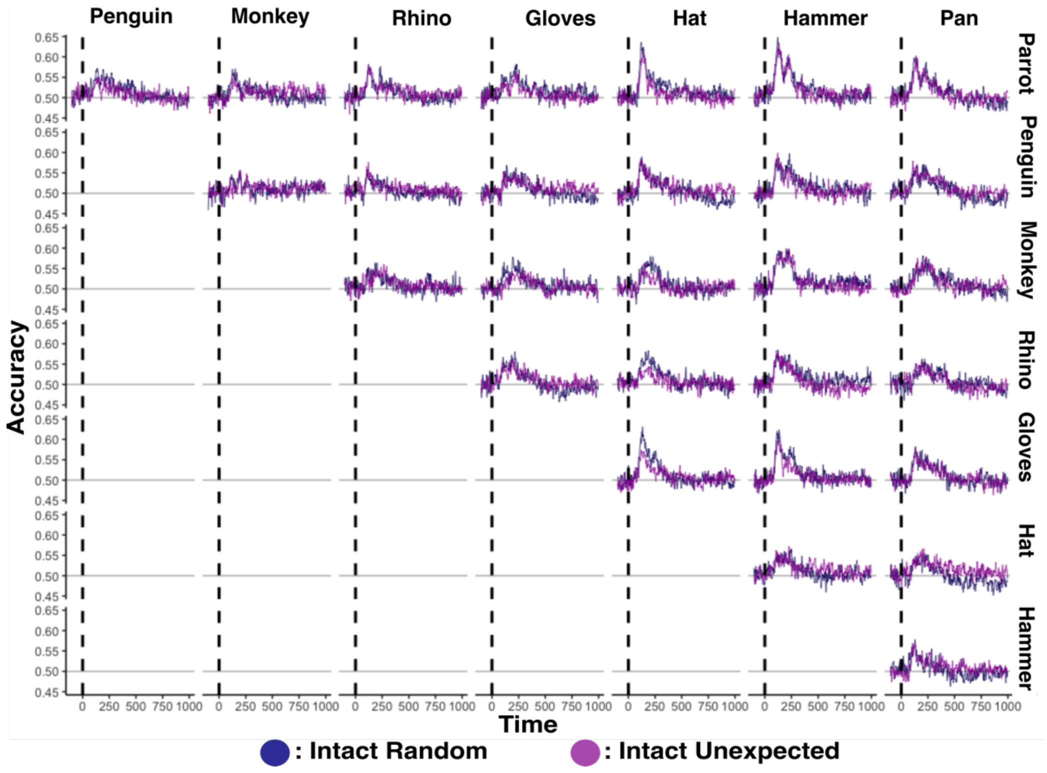

Supplementary Figure 2: *Pair-wise decoding accuracy for all stimuli included in decoding analyses comparing intact random to intact unexpected image (see Figure 5). The magnitude of difference between random and unexpected stimuli does vary across different stimulus pairs. Specifically, this effect is most prominent in comparisons between animate and inanimate stimuli (e.g., Hat vs. Rhino), but also is present in comparisons between different inanimate objects (e.g., Hammer vs. Gloves) and between different animate objects (e.g., Penguin vs. Parrot). This suggests that the reported key effect is not driven solely by differences in a specific pair-wise comparison or by differences in pair-wise accuracy across different stimulus categories.*

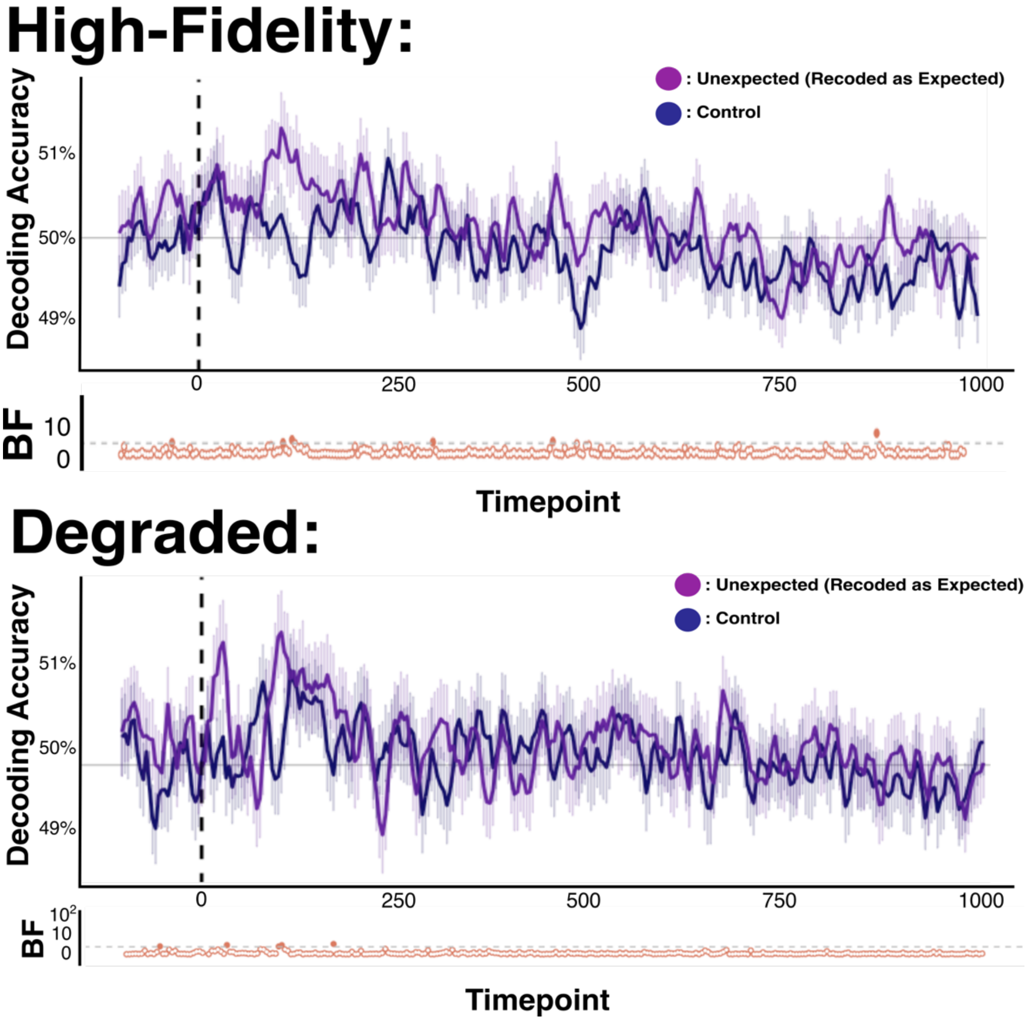

Supplementary Figure 3: *Decoding accuracy for unexpected (re-coded as expected) stimulus presentations. Plots show decoding accuracy for cases in which the unexpected stimuli were degraded or high-fidelity, respectively. In both cases, unexpected stimuli were re-coded as the high-fidelity version of the expected stimulus. Chance performance (50%) is denoted in grey and stimulus onset is denoted by the dotted vertical line. Bayes factors for each relevant decoding model comparison are reported in lower panels. Dotted grey lines mark BF = 3 (the boundary for moderate evidence). Dots which are coloured represent comparison yielding BF >3. These results trends are similar to the effects found when data from these conditions are considered together.*
